# The Ansell Fortune

**Published:** 1914-01-03

**Authors:** 


### The Ansell Fortune.

Through the death of Mr. J. C. Ansell, at the age of seventeen, the sum of £263,000 becomes available for a long list of important charities, of which the bequests to hospitals are: £50,000 to King Edward's Hospital Fund; £10,000 each to Charing Cross Hospital, Guy's Hospital, the London Hospital, the Middlesex Hospital, St. Bartholomew's Hospital, St. George's Hospital, St. Thomas's Hospital, and Westminster Hospital; and £4,000 each to King's College Hospital, University College Hospital, the Royal Free Hospital, the Great Northern Central Hospital, the Cancer Hospital, Fulham road, the Brompton Hospital for Consumption and Diseases of the Chest, City of London Hospital for Diseases of the Chest, Royal Hospital for Diseases of the Chest, London Fever Hospital, London Lock Hospital and Rescue Home, National Hospital for Diseases of the Heart, Queen Charlotte's Lying-in Hospital, Royal London Ophthalmic Hospital, Royal Orthopædic Hospital, Hospital for Diseases of the Throat, Nose, and Ear, Royal Hospital for Children and Women, Hospital for Sick Children, Great Ormond Street, and the Victoria Hospital for Children. The ultimate residue of the estate is to be held in trust for, and in such proportions for, such London hospitals, whether beneficiaries under the above distribution or not, as the trustees shall in their sole discretion think fit.

